# Poor Mental Health in Middle-Aged and Older Adults with Cognitive Difficulties

**DOI:** 10.1093/geroni/igaf122.3995

**Published:** 2025-12-31

**Authors:** Zoe Fagan, Amy Fiske

**Affiliations:** West Virginia University, Morgantown, West Virginia, United States; West Virginia University, Morgantown, West Virginia, United States

## Abstract

Cognitive difficulties and depressive symptoms are interrelated in older adults. There is some evidence that depression may be a prodromal symptom of dementia (Brommelhoff et al., 2009). In addition, depression may arise in the early stages of Alzheimer’s Disease as an emotional reaction to the awareness of loss of cognitive abilities (Rosanna et al., 2022). The primary goal of this analysis was to examine the association between cognitive difficulties and mental health in older adults in a large national dataset, and to test whether the relation can be explained by prior depressive disorder diagnosis. An additional aim was to test possible predictors of poor mental health among older adults who endorsed thinking or memory problems. The study involved analyzing data (N = 133,857 ) from the 2023 wave of the Behavior Risk Factor Surveillance System (BRFSS). Participants included adults ages 45-80 + (mean age= 55.8). Hypotheses were tested using a series of hierarchical linear regression models. Demographics included age, sex, and education. Independent variables included self-reported cognitive difficulties and life-time history of depressive disorder. Cognitive difficulties were significantly associated with the number of days of poor mental health, controlling for demographics as well as history of depression, F (5, 130,674)= 8749.54, p < 0.01, change in R2 = .034. These findings suggest a unique role for cognitive difficulties in explaining mental health status. Future studies should focus on understanding mental health outcomes in older adults with cognitive difficulties, and developing interventions to address these issues.

